# PSMD12 promotes the activation of the MEK-ERK pathway by upregulating KIF15 to promote the malignant progression of liver cancer

**DOI:** 10.1080/15384047.2022.2125260

**Published:** 2022-09-22

**Authors:** Hanpu Zhang, Chenyuan Li, Shichong Liao, Yi Tu, Shengrong Sun, Feng Yao, Zhiyu Li, Zhong Wang

**Affiliations:** a Department of Breast and Thyroid Surgery, Renmin Hospital of Wuhan University, Wuhan, P. R. China; b Department of Colorectal Surgery, the First Affiliated Hospital, Zhejiang University School of Medicine, Hangzhou, China

**Keywords:** PSMD12, KIF15, MEK, ERK, hepatocellular carcinoma

## Abstract

The tumor recurrence and drug resistance of hepatocellular carcinoma (HCC) threatened patients a lot. The mechanism should be further explored. The information of expression status and survival were available in public databases. The Western blot and immunohistochemistry staining displayed the level of related proteins. CCK-8, colony-formation assays, transwell assay and wound healing assay were performed to illustrate the ability of tumor growth, invasion and migration. In vivo model was established to verify our cell experiments. In our study, we revealed that proteasome 26S subunit, non-ATPase 12 (PSMD12) was high expressed in HCC tissues and positive related to the survival. *In vitro* experiments suggested that PSMD12 knockdown attenuated tumor cell growth, invasion and migration. Moreover, PSMD12 interference blocked the activation of MEK-ERK pathway. The ERK inhibitor could alleviate the tumor-promoting effect in PSMD12-overexpression cells. In addition, kinesin family member 15 (KIF15) was also observed to be highly expressed in HCC and be harmful to the survival. The public database, the images of immunohistochemistry and the western blot illustrated that PSMD12 and KIF15 was positive correlated. KIF15 knockdown impaired tumor progression and tumor-promoting effect of PSMD12. The xenograft models supported the results of cell experiments. In conclusion, PSMD12 could activated MEK-ERK pathway via KIF15 upregulation, thereby promoting tumor progression.

## Graphical abstract

**Figure:**
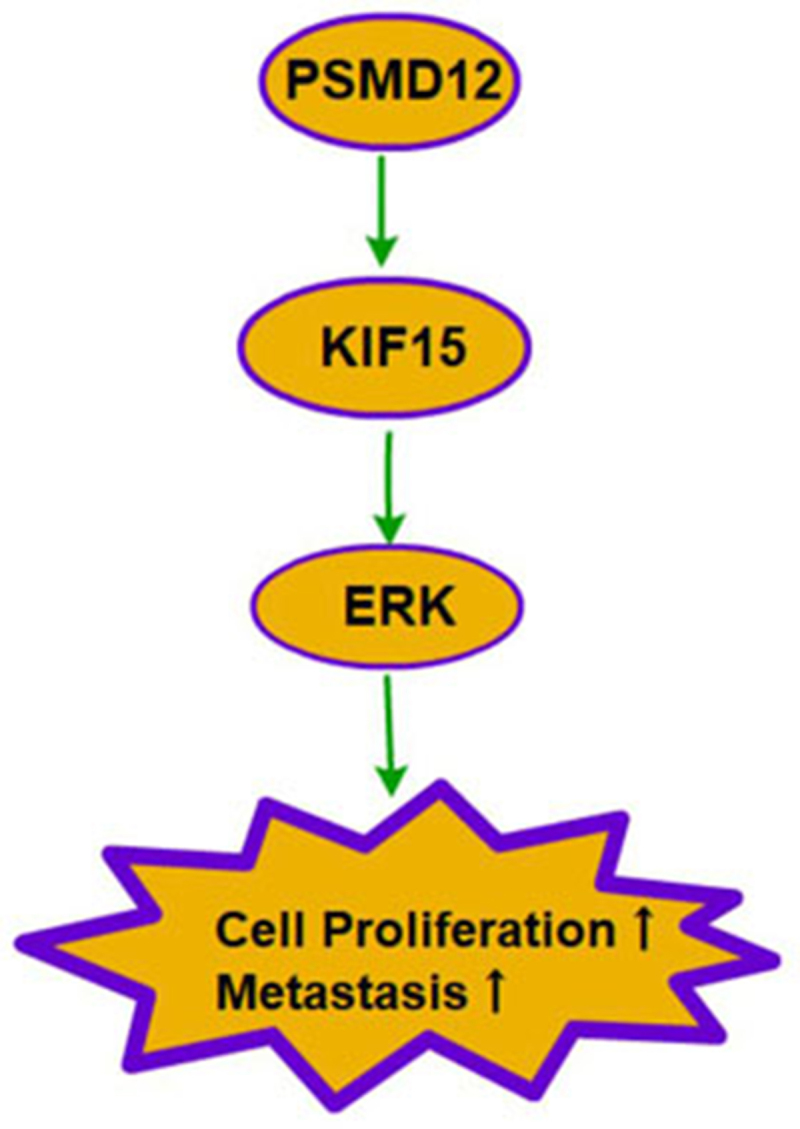

## Introduction

Primary hepatocellular carcinoma (PHC) is one of the most frequent malignant tumor types in the world, with high morbidity, recurrence, metastasis and mortality. PHC is mainly divided into hepatocellular carcinoma (HCC), intrahepatic cholangiocarcinoma (ICC), and mixed cancer.^1^ Among them, HCC is the most common type and accounts for more than 90% of new cases of liver cancer each year.^2^ Although surgical resection, liver transplantation, chemotherapy, radiation therapy, and some molecular targeted therapies can alleviate the condition and prolong the survival time of HCC patients, the treatment effect of patients with advanced HCC has obvious individual differences, and the curative effect is still very poor.^3^ The main reason is the tumor recurrence and the drug resistance of HCC cells. Therefore, there is an urgent need to find new cancer-promoting genes of liver cancer to help predict the prognosis of liver cancer patients and provide potential therapeutic targets.

Proteasome 26S subunit, non-ATPase 12 (PSMD12, also known as RPN5) is a non-ATPase subunit of the 19S regulator of the 26S proteasome complex.^4^ PSMD12 participates in several biological processes by removing damaged intracellular substructures or misfolded proteins to mediate the internal protein balance, thereby regulating the cell cycle, apoptosis or repairing DNA damage.^5,6^ It has been shown that deletion of PSMD12 could influence the function of the ubiquitin–proteasome system (UPS), consequently causing neurodevelopmental disorders.^5^ In recent years, the function of PSMD12 in tumor progression has gradually been reported; in prostate cancer, PSMD12 enhances the enzalutamide resistance of tumor cells.^7^ Moreover, PSMD12 promotes the growth of breast cancer cells.^8^ In addition, a recent study showed that PSMD12 increases Nrf2 expression, thereby promoting the progression of glioma.^9^

Kinesin family member 15 (KIF15, also known as hklp2) is a member of the kinesin superfamily of proteins (KIFs), which take part in several cell processes, including mitosis and meiosis, by driving microtubule-dependent plus-end motion.^10,11^ Emerging evidence indicates that KIF15 is involved in the regulation of various cell processes, such as proliferation, apoptosis and differentiation, and plays a crucial role in the occurrence and development of a variety of malignant tumors.^10,12–14^ In addition, KIF15 has been proposed to promote malignant tumor progression or induce tumor cell resistance to radiotherapy by regulating the MEK-ERK signaling pathway in pancreatic cancer, bladder cancer and colorectal cancer.^12,15,16^ In addition, it has also been found that KIF15 can promote an increase in the cancer stem cell (CSC) phenotype in HCC through the imbalance of reactive oxygen species (ROS) mediated by phosphoglycerate dehydrogenase (PHGDH), thereby promoting tumor progression.^13^ Nevertheless, the specific mechanism by which KIF15 affects the progression of HCC still requires additional exploration.

Here, through bioinformatics analysis, immunohistochemical staining, cellular experiments and the construction of an animal model, we first found that PSMD12 is an oncogene in HCC that can promote the progression of the malignant phenotype of tumor cells. In addition, we found that PSMD12 can activate the MEK-ERK signaling pathway by upregulating the expression of KIF15, which is the molecular mechanism by which PSMD12 promotes the malignant progression of tumor cells.

## Results

### PSMD12 is highly expressed in liver tumor tissue and closely related to the poor prognosis of patients with liver cancer

**Figure 1.**
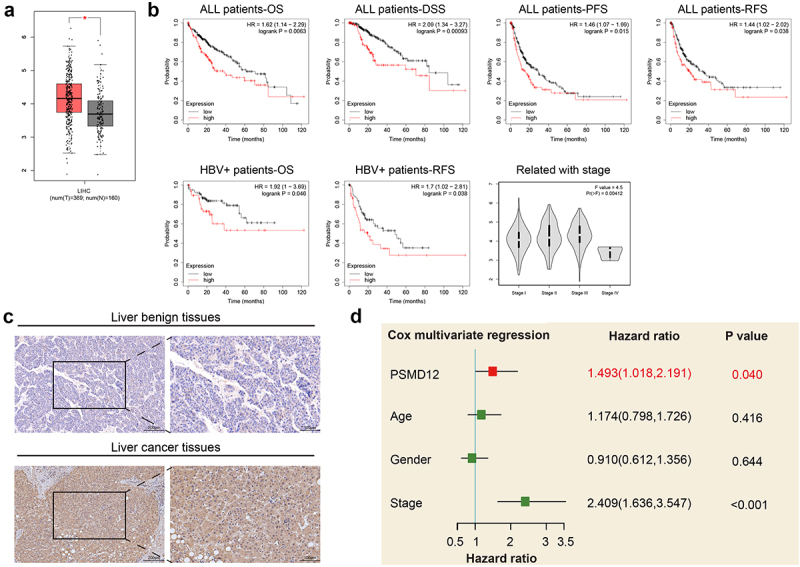
**PSMD12 was highly expressed in liver cancer tissues and indicated a worse prognosis**. (a) PSMD12 was highly expressed in liver cancer tissues (N = 369) compared with normal liver tissues (N = 160) according to analysis of data from TCGA database. (b) Kaplan–Meier analysis of all liver cancer patients and liver cancer patients infected with HBV according to PSMD12 expression from the TCGA database indicated that high expression of PSMD in liver cancer indicated a worse prognosis. Meanwhile, the expression of PSMD12 was positively associated with the tumor stage of liver cancer. (c) Expression of PSMD12 in liver cancer tissues and normal tissues by immunohistochemistry (magnification ×200) (d) According to multivariate Cox regression analysis, PSMD12 was independently associated with poor prognosis after adjustment for age, sex and tumor stage.

To explore the functions of PSMD12 in liver cancer, we analyzed the expression level of PSMD12 in tumor tissues and normal liver tissues in TCGA database. We found that the expression of PSMD12 was significantly increased in the tumor tissues compared with normal tissues (Figure 1a). The relationship between the expression of PSMD12 mRNA and the prognosis of liver cancer patients was analyzed through the Kaplan–Meier plotter online database. The results showed that the overall survival (OS), progression-free survival (PFS), recurrence-free survival (RFS) and disease-specific survival (DSS) times of patients with high PSMD12 expression were shorter than those of patients with low PSMD12 expression, indicating poor prognosis. In patients with positive hepatitis B virus, it was also found that high expression of PSMD12 was closely related to lower OS and PFS times. Moreover, the expression of PSMD12 was positively correlated with the higher-grade tumor stage of the patient, and the results were statistically significant (Figure 1b). In addition, the expression of PSMD12 was detected in normal liver tissues and liver cancer tissues. We found that PSMD12 expression was obviously higher in tumor tissues than in normal tissues (Figure 1c). Furthermore, multivariate Cox regression analysis indicated that PSMD12 expression was an independent risk factor for the prognosis of liver cancer patients (HR = 1.493, 95% CI: 1.018–2.191, P = .04) (Figure 1d).

### Knockdown of PSMD12 inhibits the growth of HCC cells in vitro

**Figure 2.**
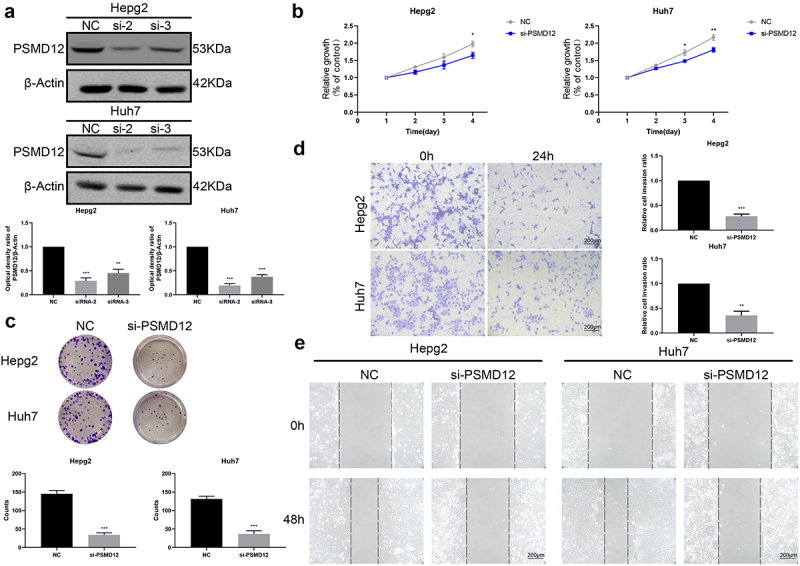
**Inhibition of PSMD12 affects cell proliferation and invasion in vitro**. (a) Knockdown efficiency of PSMD12 in HepG2 and Huh7 cells by western blot analysis. (b) Cell Counting Kit-8 (CCK-8) and (c) colony-forming assays were used to detect the cell viability of HepG2 and Huh7 cells cotransfected with PSMD12 siRNA. (d) Transwell and (e) scratch wound healing assays were performed to determine the motility of HepG2 and Huh7 cells cotransfected with PSMD12 siRNA (magnification 200×).

To further evaluate the effect of PSMD12 on the biological functions of HCC cells, we used siRNA to knockdown PSMD12 expression in HepG2 and Huh7 cells. Western blotting showed that both siRNAs decreased the expression level of PSMD12 in HepG2 and Huh7 cells, but si-2 was more efficient, reducing PSMD12 expression by at least 80% (Figure 2a). Therefore, we chose the PSMD12-homo2 siRNA sequence to silence the PSMD12 gene in liver cancer cells in subsequent biological experiments. We performed CCK-8 and plate colony formation assays to detect cell growth in HepG2 and Huh7 cell lines. The results showed that PSMD12 downregulation significantly decreased the proliferation of HCC cells (Figure 2b, c). Moreover, we observed that knockdown of PSMD12 obviously inhibited the invasion and migration of HCC cells via Transwell and wound healing assays (Figure 2d, e). Thus, PSMD12 could promote the proliferation and invasion of HepG2 and Huh7 cell lines in vitro.

### The regulatory function of PSMD12 in the proliferation and invasion of HCC cells relies on the MEK-ERK pathway

**Figure 3.**
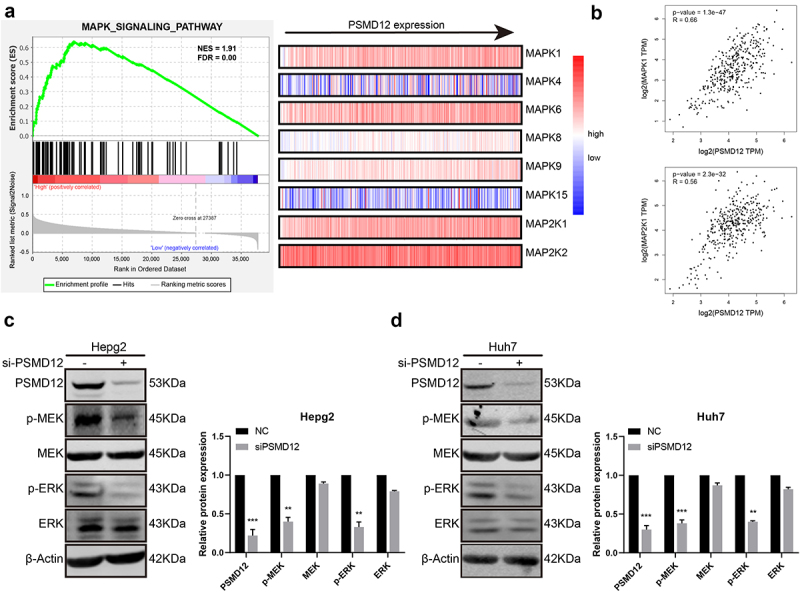
**PSMD12 promotes the activation of the MEK-ERK signaling pathway in liver cancer cell lines**. (a) PSMD12 expression was associated with the gene expression of the MEK-ERK signaling pathway through GSEA. (b) PSMD expression was positively associated with MAPK1 and MAP2K1 expression. (c) and (d) Inhibition of PSMD12 significantly downregulated the activation of the MEK-ERK signaling pathway in liver cancer cells.

**Figure 4.**
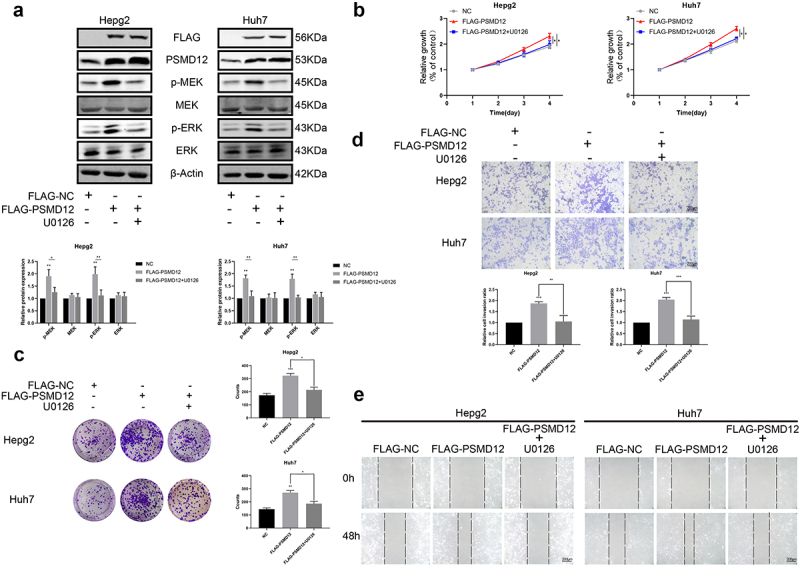
**PSMD12 promotes the progression of liver cancer cells by the MEK-ERK pathway**. (a) Levels of p-MEK, MEK, p-ERK, and ERK proteins in PSMD12-overexpressing cells with or without U0126 (2 μM, 18 H) were detected using western blotting. (b) Cell Counting Kit-8 (CCK-8) and (c) colony-forming assays were used to detect the viability of PSMD12-overexpressing cells with or without U0126. (d) Transwell and (e) scratch wound healing assays were conducted to determine the motility of PSMD12-overexpressing cells with or without U0126.

We performed GSEA based on TCGA to further explore the regulatory mechanism of PSMD12 on the proliferation and invasion of liver cancer. As shown in the figures, there was a strong correlation between the MAPK pathway and PSMD12 expression. The mRNA expression of PSMD12 was closely related to MAPK1 and MAP2K1, which indicates that PSMD12 is most likely to regulate tumor progression by the ERK pathway. (Figure 3a, b). Western blot analysis indicated that downregulation of PSMD12 highly decreased the expression of p-MEK and p-ERK in HepG2 and Huh7 cells, which was consistent with previous results (Figure 3c, d). In addition, we applied the FLAG-PSMD12 plasmid to increase the expression of PSMD12. As shown in Figure 4a, transfection with the plasmid significantly increased the expression of PSMD12 and markedly enhanced the activation of the MEK-ERK pathway. The proliferation and invasion abilities of HepG2 and Huh7 cells were also highly promoted (Figure 4b, c, d, e). Interestingly, after treatment with U0126, which is an efficient inhibitor of the ERK pathway, we observed that as the activation of the ERK signaling pathway was inhibited, the increase in the malignant phenotype of HCC cells induced by the overexpression of PSMD12 was also significantly downregulated (Figure 4a, b, c, d). Therefore, these results indicate that PSMD12 promoted the activation of the MEK-ERK pathway, thereby increasing the progression of HCC cells.

### PSMD12 regulates the expression of KIF15 and is positively related to KIF15

**Figure 5.**
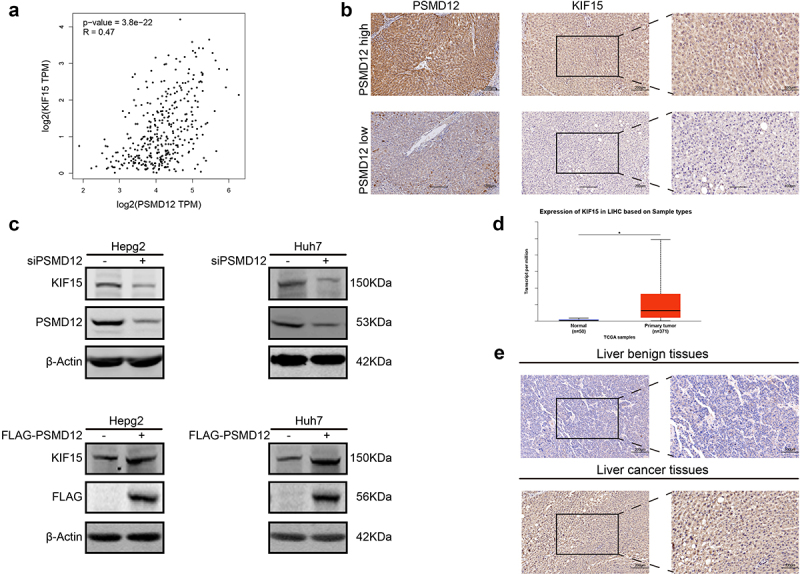
**PSMD regulates the expression of KIF15 in liver cancer cells**. (a) Correlation analysis of mRNA expression of KIF15 and PSMD12 in TCGA database. (b) Immunohistochemistry staining of KIF15 in liver cancer tissues with high/low expression of PSMD12. (c) Levels of KIF15 proteins in PSMD12 knockdown and PSMD12-overexpressing cells through PSMD12 siRNA and overexpression plasmids were detected by western blot analysis. (d) KIF15 was highly expressed in liver cancer tissues (N = 371) compared with normal liver tissues (N = 50) according to analysis of data from TCGA database. (e) Expression of KIF15 in liver cancer tissues and normal tissues by immunohistochemistry (magnification ×200).

We have proven that PSMD12 promotes the malignant progression of HCC cells via the MEK-ERK pathway. However, the specific molecular mechanism was unclear. KIF15 is a novel tumorigenic protein involved in the regulation of cancer cell biological functions. Accumulating evidence indicates that KIF15 enhances the activation of the MEK-ERK pathway in several types of tumors. Therefore, we explored whether PSMD12 participated in the regulation of KIF15. First, based on the RNA-seq data from TCGA-LIHC, we found that the mRNA expression of PSMD12 was significantly correlated with KIF15 (Figure 5a). Subsequently, we performed IHC to detect the expression of KIF15 and PSMD12 in liver cancer tissues. The result was consistent with TCGA data analysis, and KIF15 expression was highly increased in liver cancer tissues with high levels of PSMD12 (Figure 5b). Moreover, western blotting was performed to detect the expression of PSMD12 and KIF15 protein after knockdown or overexpression of PSMD12 in HepG2 and Huh7 cells. We found that knockdown of PSMD12 could significantly decrease the protein level of KIF15, while overexpression of PSMD12 increased the level of KIF15 protein (Figure 5c). But the mRNA level of KIF15 did not demonstrate corresponding difference **(Supplementary Figure 1A)**. Therefore, we detected the stabilization of KIF15 after knocking down the expression of PSMD12 in HepG2. The results suggested that PSMD12-knockdown promoted the degradation of KIF15 **(Supplementary Figure 1B)**.

### KIF15 functions as an oncogenic protein in liver cancer via the MEK-ERK pathway

**Figure 6.**
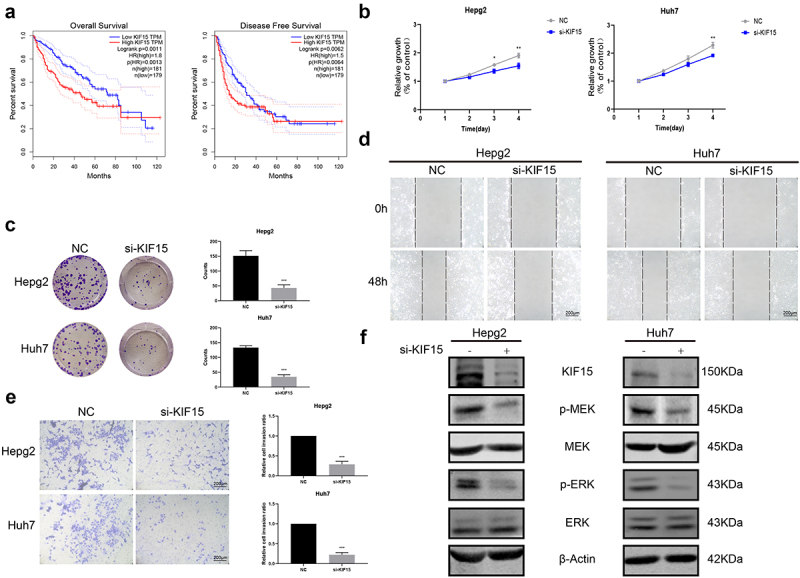
**KIF15 promotes the progression of liver cancer cells by the MEK-ERK pathway**. (a) Kaplan–Meier analysis of overall survival and disease-free survival of liver cancer patients with high or low KIF15 expression in TCGA database. (b) Cell Counting Kit-8 (CCK-8) and (c) colony-forming assays were conducted to assess the viability of HepG2 and Huh7 cells cotransfected with KIF15 siRNA. (d) Scratch wound healing and (e) Transwell assays were performed to assess the motility of HepG2 and Huh7 cells cotransfected with KIF15 siRNA (magnification 200x). (f) Levels of p-MEK, MEK, p-ERK, and ERK proteins in KIF15-downregulated cells.

To explore the functions of KIF15 in liver cancer, we assessed the expression of KIF15 mRNA in liver cancer and normal tissues from TCGA. The results showed that the mRNA expression of KIF15 in liver cancer tissues was significantly higher than that in normal liver tissues (Figure 5d). IHC analysis also yielded consistent results (Figure 5e). Moreover, based on the mRNA data and clinical information from TCGA-LIHC, we found that the expression of KIF15 mRNA was negatively related to the OS of liver cancer patients, and the prognosis of patients with high KIF15 was significantly worse than that of patients with low KIF15 (Figure 6a). To further investigate the role of KIF15 in liver cancer, we used KIF15 siRNA to decrease the expression of KIF15 protein in HepG2 and Huh7 cells and detected the biological functions of these cells. We found that treatment with KIF15 siRNA significantly downregulated the growth of HepG2 and Huh7 cells (Figure 6b, c). In addition, downregulating the expression of KIF15 also decreased the migration and invasion of tumor cells (Figure 6d, e). Interestingly, the western blot analysis results showed that knockdown of KIF15 highly inhibited the expression of p-MEK and p-ERK in HepG2 and Huh7 cells, indicating the functions of KIF15 in activating the MEK-ERK pathway in liver cancer cells (figure 6f).

### PSMD12 promotes the malignant progression of liver cancer cells by the KIF15-related MEK-ERK pathway

**Figure 7.**
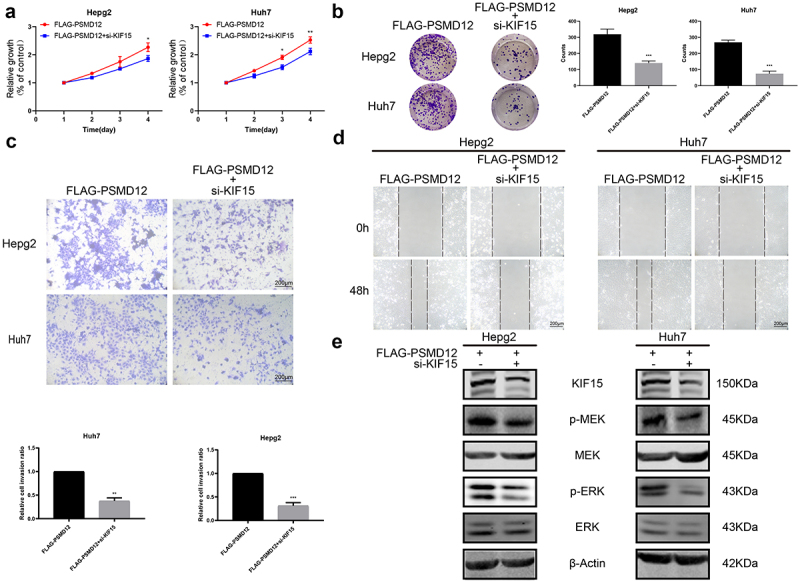
**PSMD12 activates the MEK-ERK pathway by enhancing the expression of KIF15 and promoting aggressive behaviors in liver cancer cells**. (a) Cell Counting Kit-8 (CCK-8) and (b) colony-forming assays were used to assess the cell viability of HepG2 and Huh7 cells with PSMD12 overexpression and cotransfection of KIF15 siRNA. (c) Transwell and (d) scratch wound healing assays were conducted to determine the motility of HepG2 and Huh7 cells with PSMD12 overexpression and with or without cotransfection of KIF15 siRNA. (e) Levels of p-MEK, MEK, p-ERK, and ERK proteins in liver cancer cells with PSMD12 overexpression and with or without cotransfection of KIF15 siRNA.

To investigate whether KIF15 is involved in the PSMD12-induced activation of the MEK-ERK pathway in HCC cells, we performed rescue experiments by knocking down KIF15 in PSMD12-overexpressing cells. We observed that, compared with PSMD12 overexpression alone, inhibition of KIF15 in PSMD12-overexpressing cells significantly decreased the proliferation of HepG2 and Huh7 cells (Figure 7a, b). Moreover, we also observed a similar trend in tumor cell migration and invasion (Figure 7c, d). In addition, the activation of the MEK-ERK pathway induced by PSMD12 was partly inhibited after treatment with KIF15 siRNA (Figure 7e). These results indicated that PSMD12 may regulate the expression of KIF15, thereby enhancing the activation of the MEK-ERK pathway and promoting the malignant progression of liver cancer cells.

### Knockdown of PSMD12 expression inhibits the proliferation of liver cancer in vivo

**Figure 8.**
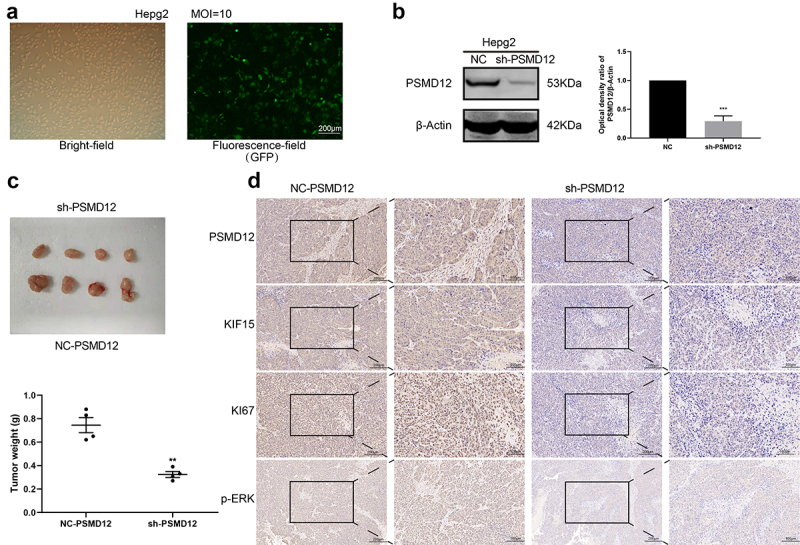
**PSMD12 enhances the tumorigenic ability of liver cancer cells in vivo**. (a) The transfection efficiency of PMD12 knockdown in HepG2 cells. (b)The knockdown efficacy of PSMD12 in HepG2 cells. (c) Xenograft pictures and the tumor weight of HepG2 cells with or without sh-PSMD12 (N = 4). (d) Immunohistochemistry staining of PSMD12, KIF15, Ki67 and p-ERK in xenograft tissues with or without sh-PSMD12.

To further determine the function of PSMD12 in liver cancer cells. We generated HepG2 cells in which PSMD12 was stably knocked down (Figure 8a, b) and injected these cells into mice to construct a xenograft model. Four weeks later, we removed the xenografts and detected the weight of these tumors. The results showed that the weight and size of xenografts in the PSMD12 knockdown group were significantly smaller than those in the NC group (Figure 8c), indicating that PSMD12 could promote the growth of tumor cells. Moreover, IHC analysis showed that knockdown of PSMD12 decreased the expression of KI67 in tumor tissue, and the p-ERK and KIF15 index of the PSMD12 knockdown group was also lower than that of the NC group (Figure 8d), which is consistent with our previous study. Therefore, these results demonstrated that PSMD12 promoted the growth rate of HepG2 cells in vivo.

## Discussion

As the most common type of primary liver cancer, HCC is still a major challenge to human health worldwide. According to the statistical evaluation of the International Agency for Cancer (IARC), by 2025, there will be more than 1 million new cases of HCC each year.^17,18^ In recent years, the prognosis of patients undergoing surgical resection has improved due to the improvement of related surgical procedures and the popularization of individualized treatment methods.^19,20^ However, due to the lack of applicable molecular markers and related therapeutic targets, liver cancer is still one of the tumor types with the highest degree of malignancy and the worst treatment effect. Therefore, there is an urgent need to find new targets in liver therapy. The function of PSMD12 as an oncogene has been reported in glioma, breast cancer and prostate cancer.^7–9^ Our study explained the function and related mechanisms of PSMD12 in liver cancer for the first time. We found that PSMD12 is a tumor-promoting gene in liver cancer, and its high expression is closely related to the poor prognosis of patients. In addition, we found that PSMD12 can promote the activation of the MEK-ERK pathway in liver cancer cells by upregulating the expression of KIF15, which is a member of the kinesin family, thereby promoting the malignant progression of tumors.

PSMD12, a non-ATPase subunit of the 19S regulator of the 26S proteasome complex, has been reported to play a crucial biological role in neurogenesis.^4,21^ In recent years, accumulating evidence has indicated the function of PSMD12 in tumor progression. In breast cancer, PSMD12 inhibits the expression of apoptosis-related genes such as TXNIP, GADD45A and GDAA45B to promote the malignant phenotype of tumor cells.^8^ In glioma, PSMD12 upregulates the expression of the transcription factor Nrf2, thereby promoting the proliferation, invasion and migration of glioma cells.^9^ Our study first reported that the expression of PSMD12 in liver cancer was significantly higher than that in normal liver tissues, and the high expression of PSMD12 is closely related to the poor prognosis of liver cancer patients. These results were similar to those of a study of PSMD12 in breast cancer and glioma, suggesting the potential role of PSMD12 as an oncogene in tumor progression.

To further investigate the functions of PSMD12 in HCC cells, we used siRNA or PSMD12 plasmids to regulate the expression of PSMD12 in HCC cells. We observed that knocking down PSMD12 in liver cancer cells can inhibit tumor cell proliferation, migration and invasion. In addition, the activation of the MEK-ERK signaling pathway was significantly downregulated in HCC cells with PSMD12 knockdown. Interestingly, after overexpression of PSMD12 in HCC cells, we found that the proliferation, migration and invasion capabilities of tumor cells were improved, and treatment with the ERK pathway inhibitor U0126 inhibited the activation of the MEK-ERK signaling pathway and reversed the malignant phenotype of tumor cells to a certain extent. The MEK-ERK pathway (also known as the ERK pathway) is an evolutionarily highly conserved signaling cascade that can transmit signals from cell surface receptors to promote cell proliferation and survival.^22–24^ The components of the ERK signaling cascade are often mutated in cancer, and inhibitor molecules targeting related kinases in the ERK pathway are gradually being used in antitumor therapy. Presently, in studies related to liver cancer, the ERK signaling pathway has been found to have a wide range of effects. In addition to promoting tumor proliferation, migration, and invasion, it has also been found to have related effects in promoting tumor cell resistance to radiotherapy and chemotherapy and immune escape.^25,26^ Our results indicated that PSMD12 can promote the malignant biological phenotype of HCC cells, and this function is achieved by activating the MEK-ERK pathway.

KIF15 is an important member of the kinesin family and a classic molecular motor that participates in many biological processes, such as cell mitosis, material transport, and cell structure formation.^27^ With the deepening of research on kinesins in tumor progression, the function of KIF15 as an oncogene to regulate tumor progression has gradually been discovered. It has been found that KIF15 can promote the malignant progression of bladder cancer, breast cancer, gastric cancer and other tumors.^28–31^ In addition, KIF15 promotes the progression of drug resistance in prostate cancer through the androgen receptor pathway.^32^ In particular, a number of studies have reported that KIF15 regulates the malignant progression of tumor cells by activating the MEK-ERK signaling pathway in pancreatic cancer, bladder cancer and colorectal cancer.^12,15,16^ In our study, we observed that the expression of PSMD12 and KIF15 had a significant correlation after analyzing the mRNA data from TCGA and the IHC results in liver tumor tissues. In addition, knockdown of PSMD12 in liver cancer cells results in a decrease in KIF15 expression, while overexpression of PSMD12 increases the protein level of KIF15. Interestingly, we found that KIF15 also activates the MEK-ERK signaling pathway in liver cancer and can promote the malignant progression of HCC cells. These results suggest that PSMD12 may promote the activation of the MEK-ERK pathway by upregulating the expression of KIF15. To test our hypothesis, we conducted a rescue experiment and observed that knockdown of KIF15 in PSMD12-overexpressing liver cancer cells can restore the progression of tumor malignancy and the activation of the MEK-ERK signaling pathway induced by PSMD12. However, there were also some limitations. The KIF15 regulation by PSMD12 was not clear in this study. Limited data were provided in Supplementary Figure 1. And further exploration was ongoing. We inferred that the regulation was correlated with the process of ubiquitylation.

In summary, our study shows that in liver cancer cells, PSMD12 promotes the expression of KIF15, thereby regulating the activation of MEK-ERK to promote malignant tumor progression. However, the specific regulatory mechanism is still unclear. What is the specific molecular mechanism by which PSMD12 regulates the expression of KIF15? What role does the ubiquitin proteasome system play in this? Therefore, further research is very necessary. In-depth study of the specific mechanism of PSMD12 in liver cancer can provide a target for the treatment of liver cancer and provide a basis for the development of new drugs.

## Materials and methods

### Data acquisition

The PSMD12 RNA-seq data and corresponding clinical information of liver cancer patients were obtained from TCGA (https://portal.gdc.cancer.gov/projects). The expression of PSMD12 in different tissues and the relationship between PSMD12 and the prognosis of liver cancer patients were analyzed by Gene Expression Profiling Interactive Analysis (http://gepia.cancer-pku.cn/) and GlioVis (http://gliovis.bioinfo.cnio.es/).

### Cell culture

The liver cancer cell lines HepG2 and Huh7 were purchased from the American Type Culture Collection (ATCC; Rockville, MD, USA). They were cultured in high glucose DMEM (HyClone) containing 10% fetal bovine serum (MRC) at 37°C and 5% CO_2_. The ERK inhibitor U0126 was purchased from MCE (HY-12031A, Shanghai, China) and diluted in dimethyl sulfoxide.

### siRNA and plasmid transfection

The siRNAs against PSMD12 (5’-GUGGUGACAAGAAGUUAGATT-3’), KIF15 siRNAs (5’-GACUGUACUUAAGGGAGCAUAUCAAdTdT-3’) and plasmids of Flag-PSMD12 and Flag-NC were obtained from the GeneChem Co. (Shanghai, China). siRNAs or plasmids and Lipofectamine 2000 (Invitrogen, Carlsbad, USA) were mixed in reduced serum medium (Opti-MEM; Gibco, USA) for subsequent experiments.

### Cell growth assay

CCK-8 assays were performed to detect cell proliferation; cells were seeded into 96-well plates (2000 cells/well). CCK-8 (Dojindo Molecular Technologies, Gaithersburg, MD, USA) solution was used according to the instructions and incubated for 2 h at 37°C. The absorbance was measured at 450 nm.

For colony-formation assays, cells were seeded in 6-well plates (400 cells/well). After 14 days, the colonies were fixed by methanol, and stained with 0.1% crystal violet for 15 minutes. The colonies were counted and photographed.

### Transwell assay

A total of 2 × 10^4^ cells were seeded in the upper chambers of 24-well plates. Serum-free medium was added in the upper chambers when complete medium was added to the lower chamber. After 24 h, the cells were fixed with formaldehyde, stained by 0.1% crystal violet, and counted.

### Wound healing assay

Liver cancer cells were seeded in six-well plates. Sterile 200 µL pipette tips were used to make the wound, and the debris was gently washed and removed with medium. The cells were cultured in medium with 1% serum for 48 h. Then we measured the narrowing of the wound via ImageJ software (NIH; National Institutes of Health).

### Western blot

RIPA (Beyotime) was used to lyse the cell samples. Proteins were separated by SDS–PAGE and transferred to nitrocellulose membranes. The membranes were blocked in 5% skim milk. Subsequently, the membranes were incubated with primary antibodies and then secondary antibodies. Antibodies against PSMD12 (Abcam ab246940), KIF15 (Proteintech 55407-1-AP), KI67 (Cell Signaling Technology 9449), ERK1/2 (Cell Signaling Technology 9695), p-ERK1/2 (Cell Signaling Technology 4370), MEK1/MEK2 (Abclonal A4868), p-MEK1/MEK2 (Abclonal AP0209), MMP9 (Cell Signaling Technology 40994), and β-actin (Cell Signaling Technology 3700) were used.

### Animal experiments

PSMD12 knockdown lentivirus purchased from GeneChem Co. (China) was transfected into HepG2 cells and selected with puromycin for four weeks. Then, the PSMD12 protein level was detected before the injection. 4- to 6-week-old female athymic nude mice (BALB/c) were randomly divided into two groups. A total of 1.5 × 10^6^ cells per milliliter of stable cells (200 µl) were subcutaneously injected. When tumor tissues grew to a suitable size, they were separated and weighed. The expression of PSMD12, KIF15, and Ki67 in xenografts was detected via IHC.

### Statistical analysis

Data are presented as the mean ± standard deviation (SD) from triplicate independent experiments. Data were assessed by SPSS version 24.0 (IBM, Armonk, NY, USA). P < .05 indicated statistically significant results.

## Data Availability

The datasets generated and analysed during the current study are available in the UALCAN (http://ualcan.path.uab.edu), GEPIA (http://gepia.cancer-pku.cn/) databases, Kaplan–Meier Plotter (https://kmplot.com/), the Cancer Genome Atlas (TCGA) data portal (https://tcga-data.nci.nih.gov/tcga/) and GEO database.

## References

[1] Bruix J, Gores GJ, Mazzaferro V. Hepatocellular carcinoma: clinical frontiers and perspectives. Gut. 2014;63(5):844–855. doi:10.1136/gutjnl-2013-306627. 24531850 PMC4337888

[2] Chidambaranathan-Reghupaty S, Fisher PB, Sarkar D. Hepatocellular carcinoma (HCC): Epidemiology, etiology and molecular classification. Adv Cancer Res. 2021;149:1–61. doi:10.1016/bs.acr.2020.10.001. PMC8796122 33579421

[3] Forner A, Reig M, Bruix J. Hepatocellular carcinoma. Lancet (London, England). 2018;391(10127):1301–1314. doi:10.1016/S0140-6736(18)30010-2. 29307467

[4] Zhang N, Osborn M, Gitsham P, Yen K, Miller JR, Oliver SG. Using yeast to place human genes in functional categories. Gene. 2003;303:121–129. doi:10.1016/S0378-1119(02)01142-3. 12559573

[5] Küry S, Besnard T, Ebstein F, Khan TN, Gambin T, Douglas J, Bacino CA, Craigen WJ, Sanders SJ, Lehmann A, et al. De novo disruption of the proteasome regulatory subunit PSMD12 causes a syndromic neurodevelopmental disorder. Am J Hum Genet. 2017;100(2):352–363. doi:10.1016/j.ajhg.2017.01.003. 28132691 PMC5294671

[6] Wang T, Jiang X, Chen G, Xu J. Interaction of amyotrophic lateral sclerosis/frontotemporal lobar degeneration-associated fused-in-sarcoma with proteins involved in metabolic and protein degradation pathways. Neurobiol Aging. 2015;36(1):527–535. doi:10.1016/j.neurobiolaging.2014.07.044. 25192599

[7] Kohrt SE, Awadallah WN, Phillips RA 3rd, Case TC, Jin R, Nanda JS, Yu X, Clark PE, Yi Y, Matusik RJ, et al. Identification of genes required for enzalutamide resistance in castration-resistant prostate cancer cells in vitro. Mol Cancer Ther. 2021;20(2):398–409. doi:10.1158/1535-7163.MCT-20-0244. 33298586 PMC7867613

[8] Du X, Shen X, Dai L, Bi F, Zhang H, Lu C. PSMD12 promotes breast cancer growth via inhibiting the expression of pro-apoptotic genes. Biochem Biophys Res Commun. 2020;526(2):368–374. doi:10.1016/j.bbrc.2020.03.095. 32222279

[9] Wang Z, Li Z, Xu H Liao, Y, Sun, C, Chen, Y, Sheng, M, Lan, Q, Wang, Z, et al. PSMD12 promotes glioma progression by upregulating the expression of Nrf2. Ann Transl Med. 2021;9(8):700. doi:10.21037/atm-21-1481. 33987398 PMC8106014

[10] Liu M, Nadar VC, Kozielski F, Kozlowska M, Yu W, Baas PW. Kinesin-12, a mitotic microtubule-associated motor protein, impacts axonal growth, navigation, and branching. J Neurosci Official J Neurosci Res. 2010;30(44):14896–14906. doi:10.1523/JNEUROSCI.3739-10.2010. PMC3064264 21048148

[11] Eskova A, Knapp B, Matelska D, Reusing S, Arjonen A, Lisauskas T, Pepperkok R, Russell R, Eils R, Ivaska J, et al. An RNAi screen identifies KIF15 as a novel regulator of the endocytic trafficking of integrin. J Cell Sci. 2014;127(Pt 11):2433–2447. doi:10.1242/jcs.137281. 24659801

[12] Wang J, Guo X, Xie C, Jiang J. KIF15 promotes pancreatic cancer proliferation via the MEK-ERK signalling pathway. Br J Cancer. 2017;117(2):245–255. doi:10.1038/bjc.2017.165. 28595260 PMC5520515

[13] Li Q, Qiu J, Yang H, Sun G, Hu Y, Zhu D, Deng Z, Wang X, Tang J, Jiang R, et al. Kinesin family member 15 promotes cancer stem cell phenotype and malignancy via reactive oxygen species imbalance in hepatocellular carcinoma. Cancer Lett. 2020;482:112–125. doi:10.1016/j.canlet.2019.11.008. 31733289

[14] Zou JX, Duan Z, Wang J Sokolov A, Xu J, Chen CZ, Li JJ, Chen HW, et al. Kinesin family deregulation coordinated by bromodomain protein ANCCA and histone methyltransferase MLL for breast cancer cell growth, survival, and tamoxifen resistance. Mol Cancer Res MCR. 2014;12(4):539–549. doi:10.1158/1541-7786.MCR-13-0459. 24391143 PMC4139106

[15] Zhao H, Bo Q, Wu Z, Liu Q, Li Y, Zhang N, Guo H, Shi B. <p>KIF15 promotes bladder cancer proliferation via the MEK–ERK signaling pathway. Cancer Manag Res. 2019;11:1857–1868. doi:10.2147/CMAR.S191681. 30881113 PMC6396666

[16] Ma Y, Zhan S, Lu H, Wang R, Xu Y, Zhang G, Cao L, Shi T, Zhang X, Chen W, et al. B7-H3 regulates KIF15-activated ERK1/2 pathway and contributes to radioresistance in colorectal cancer. Cell Death Dis. 2020;11(10):824. doi:10.1038/s41419-020-03041-4. 33011740 PMC7532977

[17] Villanueva A. Hepatocellular Carcinoma. N Engl J Med. 2019;380(15):1450–1462. doi:10.1056/NEJMra1713263. 30970190

[18] Llovet JM, Zucman-Rossi J, Pikarsky E, Sangro B, Schwartz M, Sherman M, Gores G. Hepatocellular carcinoma. Nat Rev Dis Primers. 2016;2:16018. doi:10.1038/nrdp.2016.18. 27158749

[19] Marrero JA, Kulik LM, Sirlin CB, Zhu AX, Finn RS, Abecassis MM, Roberts LR, Heimbach JK, et al. Diagnosis, staging, and management of hepatocellular Carcinoma: 2018 practice guidance by the American association for the study of liver diseases. Hepatology (Baltimore, Md). 2018;68(2):723–750. doi:10.1002/hep.29913. 29624699

[20] Llovet JM, Bruix J. Systematic review of randomized trials for unresectable hepatocellular carcinoma: chemoembolization improves survival. Hepatology (Baltimore, Md). 2003;37(2):429–442. doi:10.1053/jhep.2003.50047. 12540794

[21] Khalil R, Kenny C, Hill RS, Mochida GH, Nasir R, Partlow JN, Barry BJ, Al-Saffar M, Egan C, Stevens CR, et al. PSMD12 haploinsufficiency in a neurodevelopmental disorder with autistic features. Am J Med Genet Part B, Neuropsychiatr Genet Official Publ Int Soc Psychiatr Genet. 2018;177(8):736–745. doi:10.1002/ajmg.b.32688. PMC6261799 30421579

[22] Montagut C, Settleman J. Targeting the RAF-MEK-ERK pathway in cancer therapy. Cancer Lett. 2009;283(2):125–134. doi:10.1016/j.canlet.2009.01.022. 19217204

[23] Matallanas D, Birtwistle M, Romano D, Zebisch A, Rauch J, von Kriegsheim A, Kolch W. Raf family kinases: old dogs have learned new tricks. Genes Cancer. 2011;2(3):232–260. doi:10.1177/1947601911407323. 21779496 PMC3128629

[24] Dhillon AS, Hagan S, Rath O, Kolch W. MAP kinase signalling pathways in cancer. Oncogene. 2007;26(22):3279–3290. doi:10.1038/sj.onc.1210421. 17496922

[25] Daniel SK, Seo YD, Pillarisetty VG. The CXCL12-CXCR4/CXCR7 axis as a mechanism of immune resistance in gastrointestinal malignancies. Semin Cancer Biol. 2020;65:176–188. doi:10.1016/j.semcancer.2019.12.007. 31874281

[26] Simone V, Brunetti O, Lupo L, Testini M, Maiorano E, Simone M, Longo V, Rolfo C, Peeters M, Scarpa A, et al. Targeting angiogenesis in biliary tract cancers: an open option. Int J Mol Sci. 2017;18(2):418. doi:10.3390/ijms18020418. PMC5343952 28212293

[27] Almeida AC, Maiato H. Chromokinesins. Current Biol: CB. 2018;28(19):R1131–r1135. doi:10.1016/j.cub.2018.07.017. PMC6402541 30300593

[28] Chen Y, Fu D, Zhao H, Cheng W, Xu F. GSG2 (Haspin) promotes development and progression of bladder cancer through targeting KIF15 (Kinase-12). Aging. 2020;12(10):8858–8879. doi:10.18632/aging.103005. 32439830 PMC7288960

[29] Gao X, Zhu L, Lu X, Wang Y, Li R, Jiang G. KIF15 contributes to cell proliferation and migration in breast cancer. Hum Cell. 2020;33(4):1218–1228. doi:10.1007/s13577-020-00392-0. 32578050

[30] Ding L, Li B, Yu X, Li Z, Li X, Dang S, Lv Q, Wei J, Sun H, Chen H, et al. KIF15 facilitates gastric cancer via enhancing proliferation, inhibiting apoptosis, and predict poor prognosis. Cancer Cell Int. 2020;20(1):125. doi:10.1186/s12935-020-01199-7. 32322172 PMC7160940

[31] Zeng H, Li T, Zhai D, Bi J, Kuang X, Lu S, Shan Z, Lin Y. ZNF367-induced transcriptional activation of KIF15 accelerates the progression of breast cancer. Int J Biol Sci. 2020;16(12):2084–2093. doi:10.7150/ijbs.44204. 32549756 PMC7294947

[32] Gao L, Zhang W, Zhang J, Liu J, Sun F, Liu H, Hu J, Wang X, Wang X, Su P, et al. KIF15-mediated stabilization of AR and AR-V7 contributes to enzalutamide resistance in prostate cancer. Cancer Res. 2021;81(4):1026–1039. doi:10.1158/0008-5472.CAN-20-1965. 33277366

